# *Post-Mortem* Stability of RNA in Skeletal Muscle and Adipose Tissue and the Tissue-Specific Expression of Myostatin, Perilipin and Associated Factors in the Horse

**DOI:** 10.1371/journal.pone.0100810

**Published:** 2014-06-23

**Authors:** Philippa K. Morrison, Chen Bing, Patricia A. Harris, Charlotte A. Maltin, Dai Grove-White, Caroline McG. Argo

**Affiliations:** 1 University of Liverpool, Department of Obesity and Endocrinology, Faculty of Health and Life Sciences, Leahurst Campus, Neston, Wirral, United Kingdom; 2 Equine Studies Group, WALTHAM Centre for Pet Nutrition, Waltham-on-the-Wolds, Melton Mowbray, Leicestershire, United Kingdom; Complexo Hospitalario Universitario de Santiago, Spain

## Abstract

Obesity, a major concern for equine welfare, is highly prevalent in the leisure horse population. Skeletal-muscle and adipose tissues are important determinants of maintenance energy requirements. The myostatin and perilipin pathways play key roles in the regulation of muscle mass and lipolysis respectively and have both been associated with obesity predisposition in other mammalian species. High quality samples, suitable for molecular biology, are an essential prerequisite for detailed investigations of gene and protein expression. Hence, this study has evaluated a) the *post-mortem* stability of RNA extracted from skeletal-muscle and adipose-tissues collected under commercial conditions and b) the tissue-specific presence of myostatin, the moystatin receptor (activin receptor IIB, ActRIIB), follistatin and perilipin, genes and proteins across a range of equine tissues. Objectives were addressed using tissues from 7 Thoroughbred horses presented for slaughter at a commercial abattoir; a) samples were collected at 7 time-points from *Masseter* muscle and perirenal adipose from 5 minutes to 6 hours *post-mortem*. Extracted RN was appraised by Optical Density analysis and agarose-gel electrophoresis. b) Quantitative real time PCR and Western Blotting were used to evaluate gene and protein expression in anatomically-defined samples collected from 17 tissues (6 organs, 4 skeletal muscles and 7 discrete adipose depots). The results indicate that, under the present collection conditions, intact, good quality RNA could be extracted from skeletal-muscle for up to 2 hours *post-mortem*. However, RNA from adipose tissue may be more susceptible to degradation/contamination and samples should be collected no later than 30 minutes *post-mortem*. The data also show that myostatin and ActRIIB genes and proteins were almost exclusively expressed in skeletal muscle. The follistatin gene showed a more diverse gene expression profile, with expression evident in several organs, adipose tissue depots and skeletal muscles. Perilipin gene and protein were almost exclusively expressed by adipose tissue.

## Introduction

Obesity, having reached epidemic proportions among horses and ponies in industrialised nations, is now considered a key concern for equine welfare [1], [2]. Adipose tissue and skeletal muscle can be considered as labile reserves of energy and nutrients within the body which can be used as buffers at times of negative or positive energy balance [3]. The specific anabolic/catabolic pathways which are activated during periods of energy imbalance may be dependent on factors which regulate or modify the relative contributions of muscle or adipose tissue to whole body composition. It is widely accepted that skeletal muscle and adipose tissues engage in cross-talking pathways which ensure that they work in synergy to conserve energy balance and whole body homeostasis [4], [5]. The cross-talk between skeletal muscle and adipose tissue is achieved through the synthesis and secretion of a variety of signalling factors and hormones respectively termed myokines and adipokines.

Muscle and adipose tissues act and interact dynamically to promote energy homeostasis but in states of active weight gain/loss, homeostasis is over-ridden and the relative contributions of these tissues to body composition are altered. Two proteins have attracted increasing interest in the regulation of tissue reserves; myostatin, which regulates reserves of metabolically active muscle [6]; and perilipin, which regulates intra-cellular lipolysis [7].

Myostatin, a member of the transforming growth factor-beta super-family and one of the first myokines to be recognised, has been widely characterised as a potent negative regulator of skeletal muscle mass [8], [9]. It is secreted from skeletal muscle cells into the circulation [10], [11] and acts by binding to the activin type II receptor (ActRIIB), leading to a negative impact on muscle mass, while the circulating protein follistatin binds to, and inactivates myostatin [12].

To date, loss-of-function mutations in the myostatin gene have been associated with a dramatic increase in skeletal muscle mass in a number of mammalian species [13]–[15]. Furthermore, actions of this circulating growth factor are not restricted to muscle alone. Murine and human studies have clearly implicated myostatin in the development of obesity [16]–[18]. The myostatin protein has been detected in skeletal muscle from Thoroughbred and Kiso-uma horses [19] and polymorphisms in the equine myostatin gene have also been linked with optimal race distance in Thoroughbred horses [20]. However, despite these findings, the extent of expression of myostatin across a range of body tissues has yet to be established for the horse.

Perilipin is a complex protein which is localised to the surface of intra-cellular lipid droplets. *In vivo*, perilipin can prevent lipolysis by blocking the entry of lipases to lipid droplets [21]. In the presence of lipolytic stimuli, perilipin is phosphorylated by protein kinase A, permitting the initiation of lipolysis by the translocation of hormone sensitive lipase (HSL) to the surface of the lipid droplet. Perilipin is pivotal in governing body fat stores. Several polymorphisms of the perilipin gene have been associated with obesity and weight-loss phenotypes in humans [22], [23]. Further, the magnitude of perilipin gene expression is positively associated with obesity in humans [24], [25]. To date, perilipin expression has not been evaluated in the horse.

In order to conduct molecular and mechanistic studies, high quality samples are essential to provide RNA and proteins suitable for analysis. Collection of such samples is relatively straight forward in laboratory studies, but this is not the case for large animals such as equines, where biopsy techniques can only access superficial tissue. Hence it is critically important to develop and validate methods for the collection of high quality samples *post-mortem* prior to undertaking molecular type studies.

It is known from studies in pigs and cattle that increasing *post-mortem* interval negatively impacts on the purity and integrity of RNA [26], [27]. Therefore, before potential roles for the myostatin pathway and perilipin can be explored in the horse, it is important to first establish that good quality, intact RNA can be practically extracted from skeletal muscle and adipose tissue samples collected from the horse under commercial conditions. Further, the extent and anatomical distribution of gene and protein expression of perilipin, myostatin and associated factors remains to be established for horses.

The aims of this study were to characterise the time-course of RNA degradation in equine *Masseter* muscle and perirenal adipose tissues *post-mortem* and then to demonstrate the presence and evaluate the expression of myostatin, the myostatin receptor (ActRIIB), follistatin (an inhibitor of myostatin receptor binding) and perilipin across a spectrum of body tissues.

## Materials and Methods

### Animals and Tissue Collection

Tissues from seven mature, Thoroughbred horses were obtained *post-mortem*. All animals were in good general health and were presented for slaughter for reasons unrelated to this study (Table 1). The horses were slaughtered in a commercial abattoir (LJ Potters, Taunton, Somerset) for non-research purposes in accordance with EU legislations EC 852/2004, 853/2004 and 854/2004 on several dates between March and June 2012. Although animal procedures did not constitute an experiment as defined under the Animals (Scientific Procedures) Act 1986, all work was approved by the University of Liverpool’s Veterinary Research Ethics Committee.

**Table 1:** Phenotypic descriptors for the 7 Thoroughbred horses used in this study.

	Horse No.	Gender	Age (Years)
**Objective 1:**	1	Gelding	11
RNA time course study	2	Mare	5
	3	Gelding	8
**Objective 2:**	4	Mare	12
Across body study	5	Gelding	8
	6	Mare	10
	7	Gelding	4

### Study One: Post-mortem Stability of RNA Extracted from Masseter Muscle and Perirenal Adipose Tissue

To evaluate the time course of RNA degradation *post-mortem*, samples were collected from the *Masseter* muscle and perirenal adipose tissues of 3 animals (horses 1–3, Table 1) as these depots were those most rapidly accessible following exanguination (*Masseter*, ∼2 minutes; perirenal adipose tissue 10–15 minutes). Tissue samples (*Masseter,* around 200 g; perirenal adipose tissue, around 260 g) were aseptically collected onto sterile foil and maintained at ambient temperature (∼13°C). Gross tissue samples were sub-sampled (around 5 g) for subsequent evaluation at 5 minutes (*Masseter* muscle only), 20, 30, 40, 60, 90, 120, 240 and 360 minutes *post-mortem* using sterile equipment. All sub-samples were macerated, snap frozen in liquid nitrogen and stored at −80°C prior to RNA extraction.

### Study Two: Tissue Specific Gene Expression of Myostatin, ActRIIB, Follistatin and Perilipin

To evaluate anatomical differences in gene and protein expression throughout the body, a total of 17 samples were collected from 4 carcasses (horses 4–7, Table 1). From each carcass, six body organs, plus seven anatomically-discrete adipose depots and four skeletal muscles were sampled (Table 2). Strict anatomical descriptors were used to ensure that tissue samples were collected from the same site in each animal (Table 2). Tissue samples were obtained as rapidly as possible *post mortem* (organs and adipose tissues, within 30 minutes; skeletal muscles within 1 hour), using sterile equipment. All samples were macerated and snap frozen in liquid nitrogen before being stored at −80°C pending RNA and protein extraction.

**Table 2:** Specific anatomical descriptors used to locate the tissue collection points for the 6 visceral organs, 7 regionally discrete adipose tissue depots and 4 skeletal muscles sampled from horses used in the second objective.

Tissue		Anatomical descriptors for sample sites
Visceral organs	Myocardium	∼2 cm^3^ square, full thickness section, lateral wall of left ventricle midway between coronary groove and ventricle apex.
	Lung	∼2 cm^3^ from dorsal aspect of the caudal lobe of the left lung at the intersection of the caudo-cranial and dorso-ventral midlines.
	Liver	∼2 cm^2^ full thickness section from midway along the lateral margin of the left lobe.
	Kidney	∼2 cm^3^, largely renal medulla, from the dorsal surface of the left kidney equidistant between the hillus and caudal pole.
	Stomach	∼2 cm^2^ full thickness section from the body of the stomach, midway along the greater curvature adjacent to the origin of the greater omentum.
	Spleen	∼2 cm^3^ from midpoint on the visceral surface of the intestinal lobe.
Adipose tissues	Perirenal	∼3 cm^3^, collected from the visceral aspect of the fat mass overlying the left kidney following evisceration.
	Ventro-abdominal	∼3 cm^3^, collected from the left split-carcass midline at a point equidistant between xiphisternum and pubis.
	Epicardial	∼2 cm^3^ from the coronary groove and overlying the left coronary artery
	Omental	Variable area of omentum, sufficient to harvest ∼2 cm^3^ of adipose tissue, from a region adjoining the greater curvature of the stomach and bearing visible adipose.
	Mesenteric	Variable area sufficient to harvest ∼2 cm^3^ of adipose tissue from the jejunum/proximal ileum mesenteries bearing visible adipose tissue.
	Crest	∼3 cm^3^ from the left split-carcass at the deepest part of the crest, midway between wither and poll extremities.
	Tailhead	∼2 cm3 from the subcutaneous adipose tissue overlying the gluteal muscles of the left carcass.
Skeletal muscles	*Rectus abdominus,*	∼3 cm^3^, collected from the left split-carcass midline at a point equidistant between xiphisternum and pubis.
	*Longus colli*,	∼3 cm^3^, from its severed cranial extremity in the left split-carcass.
	*Adductor*	∼3 cm^3^, collected from the centre of the exposed midline section of the muscle on the left split-carcass.
	*Pectoralis transversus*	∼3 cm^3^, collected from the exposed midline section of the muscle at a point just ∼10 cm caudal to the thoracic inlet.

Approximate target sample sizes are given. Where relevant, tissues were collected from the left side following carcass-splitting.

### RNA Extraction

Total RNA was extracted from all frozen tissue samples using TRIzol reagent (Invitrogen, Paisley, UK), in accordance with the manufacturers protocol. RNA concentration and purity was quantified spectrophotometrically (Eppendorf Biophotometer, Hamburg, Germany). To assess the purity of the extracted RNA, the ratio of optical density (OD) of the diluted RNA sample measured at wavelengths of 260 and 280 nm, provides an indication of any contamination of the RNA sample with RNase proteins. Reverse transcription (RT) was carried out in a 10 µl final reaction volume containing 0.5 µg RNA using an iScript cDNA synthesis kit (Bio-Rad Hemel Hempstead, UK). The resulting cDNA was diluted at 1∶4 and used as a template for real-time PCR analysis. Visual appraisal and quantification of 28S and 18S RNA bands was conducted using agarose gel electrophoresis.

### Agarose Gel Electrophoresis

To assess RNA integrity from horses 1–3 (study one), 10 µg RNA from *Masseter* muscle samples; 8–10 µg RNA from perirenal adipose tissue samples were mixed with loading solution (containing 500 µl formamide, 162 µl formaldehyde and 100 µl 5 X MOPS buffer) in a 1∶3 dilution, then heated at 65°C for 5 minutes before being placed on ice. Two micro-litres of loading buffer (containing 1 ml 50% sterile glycerol, 12 µl 5% bromophenol blue, 7 µl 1M NaOH and 12 µl 10 µg/µl ethidium bromide) was then added to the samples to make a final volume of 20 µl. The samples were separated by electrophoresis through a 1% agarose gel, stained with ethidium bromide and 28S and 18S band intensities were quantified (ChemiDoc XRS+ Imaging System, BioRad).

### Quantitative Real-time PCR

The expression of myostatin and four housekeeping genes previously used in other equine studies [28], [29] (GAPDH, Beta-actin, HPRT1 and RPL32) was determined in tissues from horses 1–7 (studies 1 & 2), with the expression of a further three genes (ActRIIB, follistatin and perilipin) assessed in horses 4–7 (study 2). GeNorm and Normfinder software (GenEx, Germany) was used to assess the two ‘most stably’ expressed genes to be used for normalisation. Gene expression was determined by quantitative real-time PCR performed in duplicate using the Stratagene Mx3005P detection system (Agilent Technologies, California USA). Primer sequences for all four housekeeping genes were obtained from previously published data (HPRT1 and RPL32, GAPDH [29], and beta-actin [28]) and 100% homology was confirmed by performing a basic local alignment search tool (BLAST). Primer and Taqman probe sequences for myostatin, ActRIIB, follistatin and perilipin were designed using Beacon Designer (Premier Biosoft, USA). All primers were designed to be exon-spanning. All primer/probe sets were purchased from Eurogentec (Belgium) (Table 3). Serial dilutions of pooled cDNA were used to calculate Taqman primer efficiencies. The PCR cycling conditions (using Taqman probe and primers) for the genes of interest (myostatin, ActRIIB, follistatin and perilipin) were as follows: 10 minutes at 95°C, followed by 40 cycles of 30 seconds at 95°C, 1 minute at 55°C and 1 minute at 72°C. Cycling conditions for housekeeping genes (using SYBR green method) were as follows: 10 minutes at 95°C followed by 40 cycles of 15 seconds at 95°C and 30 seconds at 60°C and ending with, 1 minute at 95°C, 30 seconds at 55°C and 30 seconds at 95°C.

**Table 3:** Nucleotide sequences of primers and probes used in the current study.

Gene	Primer	Sequence	Amplification efficiency
Beta-actin	Forward	GGACCTGACGGACTACCTC	97%
	Reverse	CACGCACGATTTCCCTCTC	
HPRT1	Forward	GGCAAAACAATGCAAACCTT	94.5%
	Reverse	CAAGGGCATATCCTACGACAA	
GAPDH	Forward	CAGAACATCATCCCTGCTTC	95%
	Reverse	ATGCCTGCTTCACCACCTTC	
RPL32	Forward	AGCCATCTACTCGGCGTCA	94%
	Reverse	TCCAATGCCTCTGGGTTTC	
Myostatin	Forward	GCAGTGATGGCTCTTTGGAAG	97.9%
	Reverse	GCATTAGAAGATCAGACTCTGTAGG	
	Probe	ACCACGCGACGACGGAAACAATCAT	
ActRIIB	Forward	GCCTCGCTGTTCGGTTTGAG	92.9%
	Reverse	GGCTCCCTCAAGCACCTCAG	
	Probe	ACCGCCGTGTGCCCACCTGC	
Follistatin	Forward	CAGTGACAATGCCACTTACGC	92.5%
	Reverse	GGTCTTCATCTTCCTCCTCTTCC	
	Probe	TGCCATGAAGGAAGCTGCCTGTCTCC	
Perilipin	Forward	GATCCCAGCCCTCCAGTACC	103.9%
	Reverse	GGACGCTGATGCTGTTCCTG	
	Probe	AGATCGCCTCTGAGCTGAAGGACACCATC	

Relative gene expression was calculated using the comparative Ct method (2^−ΔΔCt^) [30]. All gene expression data were normalised to 2 internal housekeeping genes and data from the second study are presented as relative expression with respect to the myocardial tissue.

### Protein Extraction and Western Blotting

Total protein was extracted from frozen tissues obtained from the horses 4–7 (study two) by homogenising around 100 mg of tissue in a SHE buffer (250 mM sucrose, 1 mM HEPES, 0.2 mM EDTA) containing both phosphatase and protease inhibitor cocktails (both Sigma, Poole, Dorset, UK). Protein concentration was determined by the BCA method and protein integrity was verified using standard silver staining of typical SDS gels (data not shown).

Forty five micrograms of protein extract were separated on 10% SDS-polyacrylamide gels under reducing conditions and proteins were transferred onto nitrocellulose membrane (Hybond-C Extra, Amersham Bioscience, Buckinghamshire, UK) by electroblotting. Membranes were stained in Ponceau S reversible stain to verify the success of protein transfer and then blocked for 1 hour in 5% BSA in Tris-buffered saline containing 0.1% Tween 20 (TBST). Commercially available primary antibodies (listed below) were used and were selected on the basis that they were listed as having equine cross-reactivity. They were added at the following concentrations: myostatin precursor (MSTN), 1∶250 [ab98337 Abcam, Cambridge, UK], myostatin receptor (ACTRIIB), 1∶200 [sc-25453 Santa Cruz, Dallas, Texas, USA], PLIN, 1∶200 [sc-67164 Santa Cruz], the serine/threonine Akt (AKT), 1∶2500 [#9272 Cell Signalling, Danvers, MA, USA]) in blocking buffer and incubated overnight at 4°C. The myostatin antibody detected the precursor form of the protein (43 kDa). The membranes were washed and then incubated for 1 hour with a secondary antibody (Cell Signalling) at appropriate concentrations. Signals were detected by chemiluminescence using a SuperSignal West Pico Chemiluminescent Substrate (Pierce, Rockford, IL, US) and visualised on a Molecular Imager ChemiDoc XRS+ System (Bio-Rad).

### Statistical Analysis

Statistical analyses were performed using STATA version 12.1. Non-parametric, analytical methods were employed. The Friedman test for repeated measures was used to assess the effect of increasing *post-mortem* interval on myostatin gene expression in study 1. The Wilcoxon signed ranks test was used to analyse gene expression data from study 2.

## Results

### Study One: Post-mortem Stability of RNA Extracted from Masseter Muscle and Perirenal Adipose Tissue

The purity of RNA extracted from the *Masseter* muscle remained relatively stable over the 6 hour time course evaluated. The ratio of OD for RNA extracted from muscle remained in excess of 1.8, indicating minimal protein contamination [31], for all horses at each time point (Table 4). Visual assessment of the RNA integrity confirmed that intact 28S and 18S ribosomal RNA bands were detected at all time points up to 120 minutes in all three animals (1–3). Quantification of the 28S and 18S ribosomal RNA bands demonstrated consistent 28S∶18S ratios of close to 2 at all time points up to 240 minutes *post-mortem* (Table 4). However by 360 minutes the average ratio had reduced to 1.42 and the variation was considerably increased (Table 4). These results show *Masseter* muscle is resilient to *post-mortem* RNA degradation, but samples should be obtained within 2 hours of death to ensure the extraction of good RNA for downstream molecular biology studies.

**Table 4:** RNA quality assessment by spectrophotometer (A260/A280 ratio) and 28S∶18S ratio (agarose gel electrophoresis and Chemi-Doc imaging and analysis) for *post-mortem* intervals from 5 to 360 minutes. n = 3.

Tissue	*Post-mortem* interval (minutes)	Average A260/280 ratio (standard deviation)	Average 28S∶18S ratio (standard deviation)
Massater muscle	5	2.10 (0.16)	1.95 (0.23)
	20	2.05 (0.12)	1.82 (0.07)
	30	1.98 (0.04)	1.87 (0.19)
	40	2.02 (0.08)	2.01 (0.15)
	60	1.92 (0.13)	1.98 (0.15)
	90	2.06 (0.10)	1.92 (0.19)
	120	1.99 (0.01)	1.81 (0.18)
	240	1.95 (0.03)	1.85 (0.06)
	360	1.85 (0.05)	1.42 (1.07)
Perirenal adipose tissue	20	1.71 (0.41)	1.77 (0.79)
	30	1.63 (0.42)	1.67 (0.18)
	40	1.63 (0.40)	1.48 (0.19)
	60	1.65 (0.40)	1.60 (0.64)
	90	1.63 (0.36)	1.56 (0.12)
	120	1.62 (0.42)	1.53 (0.50)
	240	1.68 (0.39)	1.28 (0.57)
	360	1.44 (0.41)	1.54 (0.09)

By contrast, in adipose tissue, the OD ratios of RNA extracted from perirenal adipose tissue were relatively lower and more variable than those obtained from *Masseter* muscle (Table 4). Mean OD ratios ranged from 1.71 to 1.68 in samples evaluated between 20 minutes and 4 hours *post-mortem* but had decreased to 1.44 by the final test at 6 hours *post-mortem* (Table 4). Visual appraisal of RNA integrity demonstrated that intact 28S and 18S ribosomal bands were visible in all horses (horses 1–3) up to 30 minutes *post-mortem*. Quantification of the 28S and 18S ribosomal bands demonstrated the average 28S∶18S ratios were 1.77 and 1.67 at 20 minutes and 30 minutes *post-mortem*, respectively, and gradually decreased down to 1.54 by 6 hours *post-mortem* (Table 4). This indicates that adipose tissue appears to be more susceptible to RNA degradation than skeletal muscle. However, intact RNA can be extracted from adipose tissue provided samples are collected up to 30 minutes *post-mortem* under conditions similar to those used in this study.

### Expression of Myostatin in Masseter Muscle and Perirenal Adipose Tissue

GeNorm and Normfinder results (Table 5) indicated that HPRT1 and Beta-actin were the most stably expressed of the housekeeping genes evaluated. On this basis, the mean Ct values of these genes were used for the normalisation of gene expression data. Friedman tests demonstrated no difference in myostatin expression across the time course in either *Masseter* muscle (*p* = 0.16) or perirenal adipose tissue (*p* = 0.96).

**Table 5:** Housekeeping gene comparison using GeNorm and Normfinder analysis.

	Study 1		Study 2	
Gene	GeNorm M value (ranking)	Normfinder SD (ranking)	GeNorm M value (ranking)	Normfinder SD (ranking)
HPRT1	1.41 (1)	0.41 (1)	1.20 (1)	0.25 (1)
B-ACTIN	1.41 (1)	0.41 (2)	1.55 (3)	1.46 (3)
RPL32	1.81 (2)	0.79 (3)	1.20 (1)	1.25 (2)
GAPDH	2.35 (3)	0.90 (4)	2.74 (4)	3.92 (4)

### Study Two: Tissue Specific Gene Expression of Myostatin, ActRIIB, Follistatin and Perilipin

For study two, RNA quality was assessed spectrophotometrically and was shown to be acceptable for the proposed study of gene expression in all tissues (Table 6). Gene expression data were normalised with respect to those for RPL32 and HPRT1. When gene expression data across the entire range of tissues sampled were assessed with GeNorm and Normfinder software, RPL32 and HPRT1 demonstrated the greatest stability of all housekeeping genes examined (Table 5).

**Table 6:** RNA quality as assessed by spectrophotometry; average A260/A280 ratios from various tissues throughout the body.

Tissue	Average A260/A280 ratio(standard deviation)
Myocardium	1.91 (0.04)
Lung	1.90 (0.03)
Liver	1.86 (0.06)
Kidney	1.85 (0.02)
Stomach	1.90 (0.03)
Spleen	1.83 (0.05)
Omental fat	1.81 (0.06)
Mesenteric fat	1.81 (0.04)
Retroperitoneal fat	1.74 (0.03)
Crest fat	1.78 (0.05)
Tailhead fat	1.73 (0.10)
Perirenal fat	1.70 (0.08)
Epicardial fat	1.74 (0.06)
Rectus abdominus	1.97 (0.14)
Longus colli	1.93 (0.18)
Adductor	1.92 (0.03)
Pectoralis transversus	1.97 (0.06)

(Study 2; n = 4).

**Figure 1:**
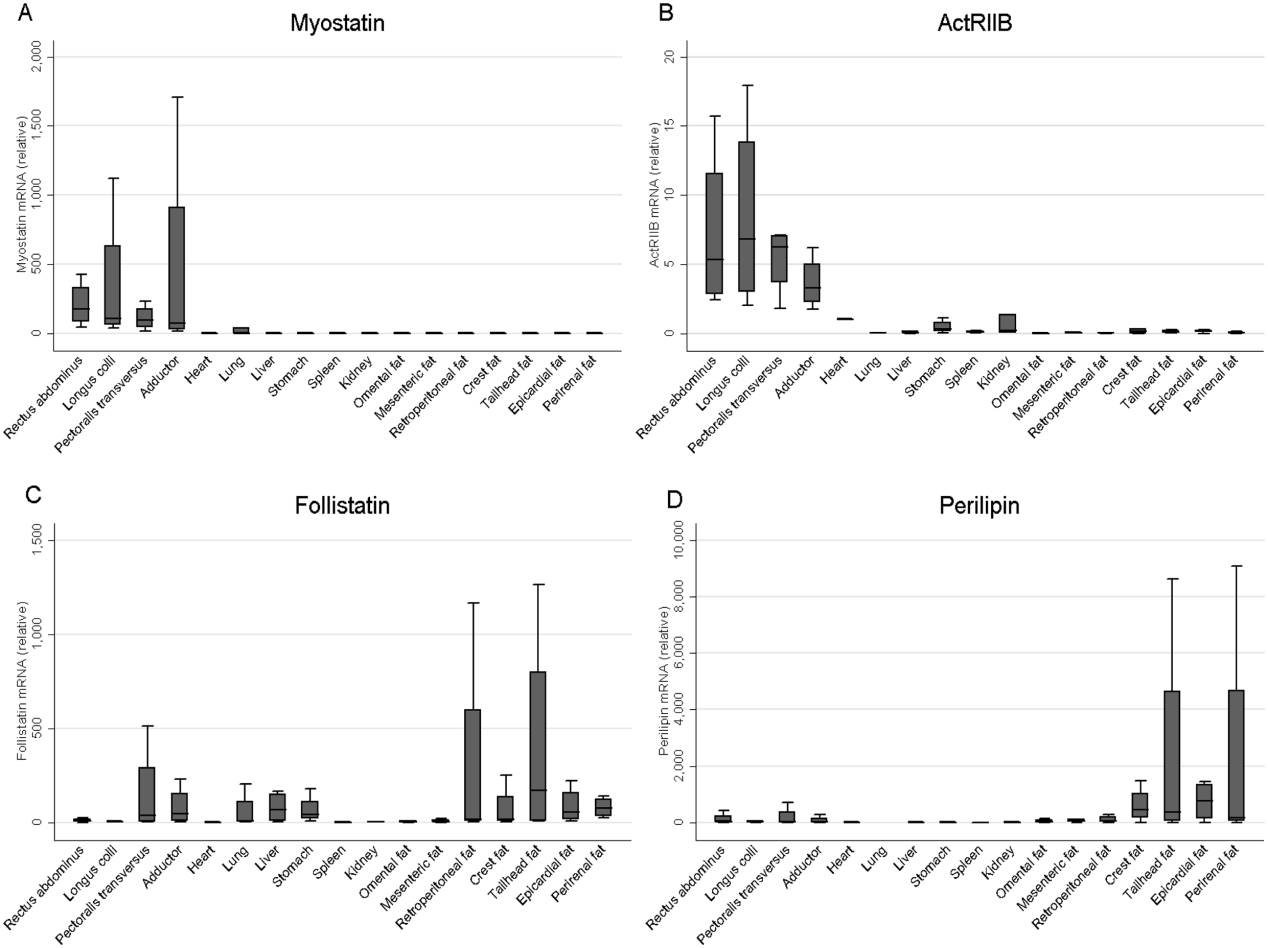
Gene expression of Myostatin, ActRIIB, Follistatin and Perilipin across a range of equine tissues as analysed by quantitative real-time PCR. Data are presented as relative expression with respect to myocardial tissue. The geometric mean of the two most stable housekeeping genes as determined by GenNorm (HPRT1 and RPL32) was used for normalisation. Relative transcript abundance is shown for a) Myostatin, b) ActRIIB, c) Follistatin, and d) Perilipin. *n* = 4.

Visual appraisal of myostatin gene expression (Figure 1a) demonstrates considerably greater expression in skeletal muscles compared to any other tissues studied (p = 0.07 for all muscles). Although relative transcript concentrations in skeletal muscle appear varied between the specific muscles sampled, differences were not statistically significant. The anatomical distribution in the relative abundance of ActRIIB mRNA was similar to that of myostatin (Figure 1b), with expression of this gene being greater in the skeletal muscles when compared to all other tissues studied (p = 0.07 for all muscles relative to cardiac tissue). Similarly, ActRIIB mRNA expression was not significantly different between the four skeletal muscles evaluated.

Follistatin mRNA expression was anatomically more diverse (Figure 1c). Whilst no significant differences were detected, follistatin gene expression appears to be greater than cardiac tissue in lung, liver, stomach (p = 0.07), a number of adipose tissue depots (retroperitoneal, crest, tailhead, epicardial and perirenal; p = 0.07), and three of the four skeletal muscles tested (*Longus colli, Pectoralis transversus* and *Adductor,* p = 0.07). Within the regional adipose tissues studied, crest, tailhead, epicardial, and perirenal samples tended to have greater follistatin expression when compared to the omental and mesenteric depots (p = 0.07).

Relative to myocardial tissue, visual appraisal suggests that perilipin mRNA expression tends to be greatest in all seven adipose tissue depots studied (*p* = 0.07) (Figure 1d). Between adipose depots, tailhead, epicardial and perirenal tended to have greater perilipin expression when compared to omental and mesenteric depots (p = 0.07), whilst retroperitoneal also had increased expression relative to omental (p = 0.07) and crest had greater expression compared to mesenteric fat (p = 0.07).

### Tissue Specific Protein Expression of Myostatin, ActRIIB and Perilipin

**Figure 2:**
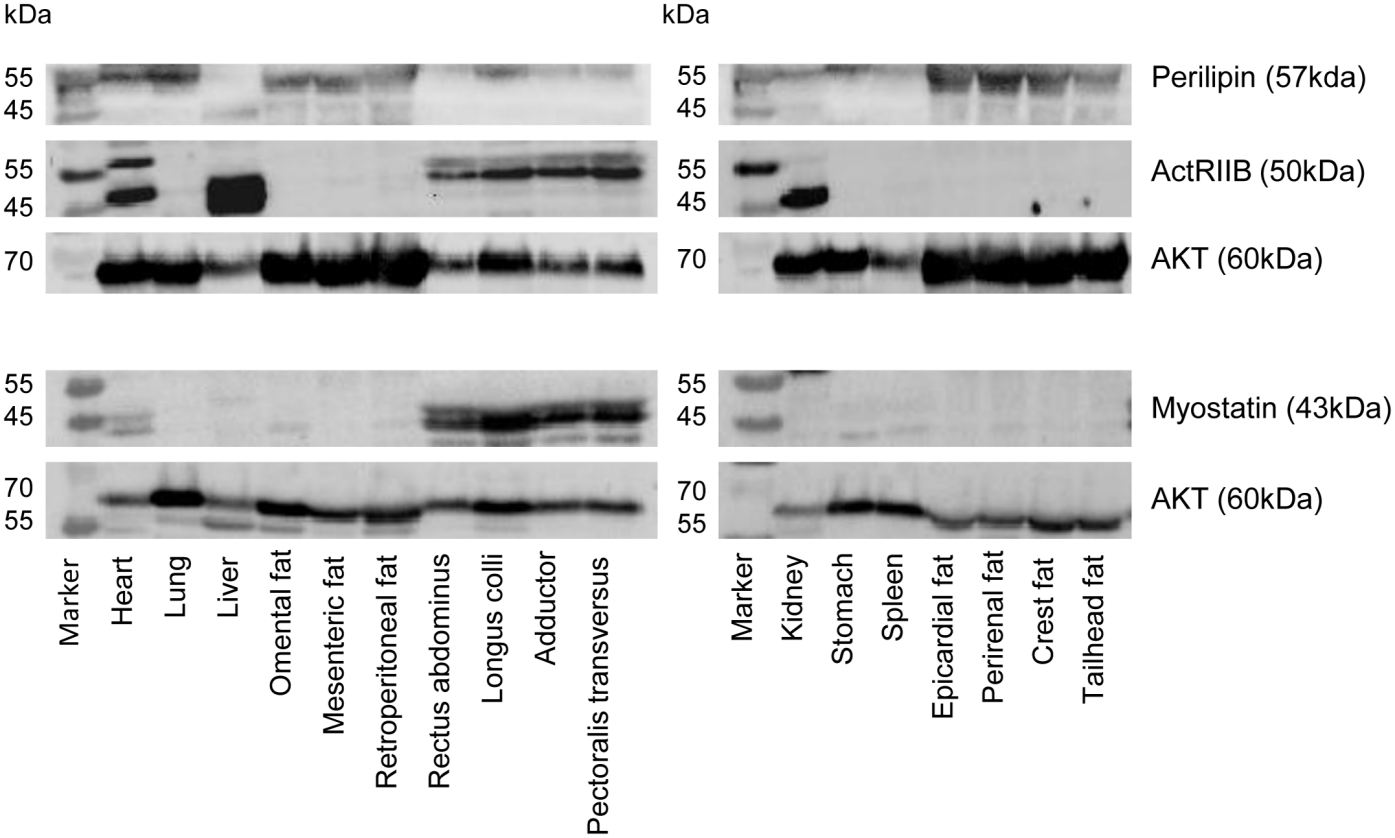
Tissue-specific protein expression of Perilipin, ActRIIB and Myostatin across a range of equine tissues. Protein expression of perilipin, ActRIIB and myostatin in a range of equine tissues was assessed by Western blot with total AKT used as a loading control. Four membranes were probed for each horse (2 for myostatin and AKT, and a further 2 for ActRIIB, perilipin and AKT) Representative blots are shown; *n* = 4. AKT loading controls are shown for each respective membrane.

Western blot analysis was used to assess protein expression of myostatin, ActRIIB and perilipin across the range of tissues collected (Figure 2). Total AKT was used as a loading control [32]. Whilst there appeared to be some non-specific binding, the myostatin (43 kDa) and ActRIIB (50 kDa) proteins were only identified in skeletal muscle samples. Perilipin protein (57 kDa) was demonstrably present in all of the studied adipose tissue depots.

## Discussion

This study establishes for the first time that it is possible to obtain samples of sufficient quality for molecular studies from the collection of *post-mortem* tissues from horses in a commercial abattoir.

By characterising the time course of RNA degradation in the immediate *post-mortem* interval, clear time constraints for the collection of *post-mortem* tissues to be used in the evaluation of gene-expression in equine skeletal muscle and adipose tissue have been described. The data indicate that while the RNA extracted from *Masseter* muscle in all horses sampled was minimally contaminated with protein (OD [A260/A280] ratio >1.8), for at least 6 hours *post-mortem,* 28S and 18S ribosomal bands were only clearly visible in all three animals for the first 2 hours following death, and average 28S∶18S ratios dropped to 1.42 by 6 hours *post-mortem*. It was noteworthy that perirenal adipose tissue appeared to be more susceptible to protein contamination. Extracting good quality RNA from adipose tissue is known to be more challenging than RNA extraction from other tissues due to the naturally high lipid content in these tissues [33], [34]. The OD (A260/A280) ratio of adipose tissue RNA remained consistently less than that of *Masseter* muscle, and whilst ribosomal RNA bands were only clearly visible up to 30 minutes *post-mortem* in all horses, average 28S∶18S ratios were 1.77 and 1.67 at 20 and 30 minutes *post-mortem,* indicating RNA remained relatively intact up to 30 minutes *post-mortem.* The gold standard 28S∶18S ratio for intact RNA is 2∶1. However it is rare to find this ratio in RNA extracted from mammalian tissues and a cut-off of 1.5 [35] and even 1.0 [36] (quantified from agarose gel electrophoresis) has been used for extracted RNA deemed to be suitable for quantitative RT-PCR. Furthermore, a recent publication that outlines criteria for publishing RT-qPCR data suggests that a 28S∶18S ratio quantified from agarose gel electrophoresis of between 1 and 2 is indicative of intact RNA [37].

Taken together, these data would suggest that the optimal windows for the collection of muscle and adipose tissue samples to ensure the extraction of good quality RNA to be used in gene expression studies are up to 120 and 30 minutes respectively. Conversely, in both the muscle and adipose tissue samples collected here, evaluation of myostatin expression in all animals seemed unaffected by *post-mortem* intervals of up to 6 hours. This could be interpreted to suggest that specific mRNAs might be variably robust. However, demonstration of the suitability of RNA extracted from tissues collected out with the defined optimal time-windows would need to be demonstrated on a gene-specific basis.

Although numerous publications use abattoir-derived, *post-mortem* tissues to describe gene-expression in large animal species, data describing the suitability of RNA obtained in this manner are sparse. Two studies have suggested that skeletal muscle RNA can remain stable up to 24 hours *post-mortem* in porcine carcasses [27] and for up to 8 days in the bovine [26]. In agreement with the current study, the bovine study also found that RNA extracted from subcutaneous adipose tissue was more susceptible to degradation than skeletal muscle, with 28S and 18S rRNA molecules remaining intact for 24 hours *post-mortem* [26]. These sampling windows greatly exceed those indicated in the current equine study and may be attributable to differences in conditions between commercial abattoir systems. The porcine and bovine studies were conducted in high throughput abattoirs where carcasses were rapidly processed and moved to cold rooms (4°C) for further sampling within 2 hours of death. This is in contrast to the low throughput system central to the current equine study and where all tissue sampling was conducted at ambient temperature (∼13°C). Clearly, the impact of environmental temperatures is likely to have important implications for the long term stability and integrity of RNA and should be considered in conjunction with the *post-mortem* interval.

The second element of this study aimed to demonstrate the tissue-specific presence of myostatin, ActRIIB, follistatin and perilipin, and is the first study to do this in equine tissues.

GeNorm and Normfinder analysis revealed that when the whole spectrum of tissues were analysed, HPRT1 expression remained stable across the spectrum of tissues in both studies. However in study two, RPL32 proved to be more stable especially across the organ tissues than beta-actin which was used for normalisation with HPRT1 in the first study (muscle and adipose tissues only). This would suggest that the same combinations of housekeeping genes are not always suitable for normalisation when a range of tissues are studied, a finding which has been demonstrated in a number of studies [38]–[40]. Hence, careful consideration must be given to ensure the stability of housekeeping genes selected for the particular tissues under consideration.

Anatomically, the expression of myostatin and its receptor (ActRIIB), both at the gene and protein transcript level would appear to be predominantly a function of skeletal muscle. Conversely, perilipin expression was primarily restricted to adipose tissue depots while the follistatin gene was more ubiquitously expressed across a range of diverse tissues. The small population size in this study combined with large differences in gene expression between animals may be accounting for the lack of statistically significant differences between tissues in the gene expression studies, however it is clear from the gene and protein expression data that there are differences between tissues and a greater study population may have increased the statistical significance.

Studies of myostatin in large animal species have generally focused on associations between myostatin gene mutations and carcass traits in breeds of cattle [41], sheep [42], and pigs [43] as an adjunct to selective breeding programs for optimal meat production. There are few studies in the horse and to date, only one report has identified myostatin precursor and mature myostatin protein expression in the skeletal muscles (*Semitendinosus, Semimembranosus, Splenius, Gluteus medius)* of Thoroughbred and Kiso-uma breeds of horses [19]. That gene and protein expression for myostatin and its receptor were largely exclusive to skeletal muscle, suggested that as reported for other species, myostatin may have an important role in equine muscle function. Notably, the gene expression of myostatin has also been reported to differ between skeletal muscle fibre types; with increased mRNA expression recorded in muscles largely composed of type II fibres [44], [45]. Although the skeletal muscles sampled in the current study were selected for their diversity of form and function and were therefore likely to vary in fibre composition, the relative extent of myostatin gene expression in the different muscles studied here did not prove significantly different and may be due to the small number of horses studied.

Although myostatin and its receptor were not detected at the gene and protein level in adipose tissues, evidence from other species suggests that myostatin can interact with adipose tissue to inhibit adipocyte differentiation *in vitro* [46], [47]. Conversely myostatin-mediated adipose: muscle cross-talk has been demonstrated to up-regulate gene-associated adipogenesis in mice [18]. The disparity of the conclusions of these studies is likely to be partially associated with the different experimental approaches used. It is possible that further studies are needed to evaluate the role of myostatin in cross-talking pathways with adipose tissues in *Equidae*.

The follistatin gene was liberally expressed across the range of tissues studied in these horses. Follistatin is a multi-functional protein, originally described as an inhibitor of follicle-stimulating hormone (FSH) secretion [48]. It has since been well characterised as a binding protein that inhibits the actions of members of the TGF-β family of signalling molecules including activin, myostatin, and bone morphogenetic proteins (BMPs) [49], [50]. Expression of the follistatin gene has been demonstrated across a wide range of human tissues [51]. Notably, this human study did not evaluate follistatin expression in adipose tissues which is one tissue in which follistatin gene was expressed in the current study. More recently, follistatin gene expression has been detected in human adipose tissues with greater expression noted in subcutaneous as opposed to visceral adipose depots [52]. These data agree with the findings of the current study where follistatin expression was notably minimal in omental and mesenteric depots, in marked contrast to the clear expression noted in the other adipose tissue depots studied. The same study also demonstrated that treatment with exogenous follistatin could promote adipogenesis in cultured human progenitor cells and could reverse adipogenic inhibition by myostatin [52]. Additionally, follistatin reversed the inhibitory effects of activin A on the differentiation of bovine pre-adiopocytes [53], whilst it was also identified that follistatin binds myostatin to a slightly lesser extent than it binds activin A [54]. Activin A has been implicated as a key player in human adipogenesis [55]. In the current study, follistatin gene expression was greater in subcutaneous depots (crest and tailhead) relative to visceral (omental) fat. Although not measured in the current study, it could be suggested that if activin A is expressed in equine adipose tissues it may associate with follistatin to aid in the regulation of adiopogenesis.

In the relatively lean horses used in the current study, perilipin (gene and protein), was almost exclusively expressed by adipose tissues, and was remarkably consistent between regional adipose depots. This contrasts with human work which indicated that perilipin gene expression was greater in subcutaneous than visceral adipose tissues [56], [57], but that the perilipin protein concentrations were similar between omental and subcutaneous fat depots [56], [57]. This may suggest that perilipin expression is subject to post-transcriptional modification. Perilipin expression has previously been shown to be modified in an obese state, with expression positively correlated to percentage body fat in human subjects [24], [25]. Conversely, it was observed that whilst perilipin gene and protein expression in subcutaneous adipose tissue was significantly decreased in obese as opposed to lean humans, perilipin mass per adipocyte was constant between obese and non-obese people [56]. In agreement with this, a further study also found that perilipin protein content was relatively decreased in subcutaneous adipose tissues from obese compared to lean women [7]. These data suggested that perilipin concentrations were positively associated with rates of basal lipolysis. These conflicting data may in part be associated with differences in the case-definitions for obesity between the two studies. Indisputably, perilipin plays a vital role in the regulation of lipolysis [58], [59] and it could be suggested that variations in perilipin expression are both the cause of the metabolic dysregulation apparent in obesity and a consequence of obesity itself. To the authors’ knowledge, this is the first study to identify perilipin expression in the horse and future studies may aid in the resolution of the precise contribution of this protein in the fat biology of the horse.

In conclusion, this study clearly demonstrated that RNA remains intact up to 2 hours *post-mortem* in equine *Masseter* muscle and up to 30 minutes *post-mortem* in perirenal adipose tissue in all three horses studied. Furthermore, the tissue distributions for myostatin, follistatin, ActRIIB and perilipin in the horse have been described. More focused research into how these factors are altered in settings of energy imbalance such as observed in obesity or weight loss are required to better understand their physiological roles.
